# Salvage Involved-Field and Extended-Field Radiation Therapy in Positron Emission Tomography–Positive Nodal Recurrent Prostate Cancer: Outcomes and Patterns of Failure

**DOI:** 10.1016/j.adro.2022.101040

**Published:** 2022-07-28

**Authors:** Adeline Pêtre, Magali Quivrin, Nathalie Briot, Jihane Boustani, Etienne Martin, Igor Bessieres, Alexandre Cochet, Gilles Créhange

**Affiliations:** a Department of Radiation Oncology, Centre Léon Bérard, Lyon, France; b Department of Radiation Oncology, Centre Georges François Leclerc, Dijon, France; c Department of Biostatistics Unit, Centre Georges François Leclerc, Dijon, France; d Department of Medical Physics and Radiation Oncology, Centre Georges François Leclerc, Dijon, France; e Department of Nuclear Medicine, Centre Georges François Leclerc, Dijon, France; f Department of Radiation Oncology, Institut Curie, Saint-Cloud, France

## Abstract

**Purpose:**

The optimal salvage pelvic treatment for nodal recurrences in prostate cancer is not yet clearly defined. We aimed to compare outcomes of salvage involved-field radiation therapy (s-IFRT) and salvage extended-field radiation therapy (s-EFRT) for positron emission tomography/computed tomography–positive nodal-recurrent prostate cancer and to analyze patterns of progressions after salvage nodal radiation therapy.

**Methods and Materials:**

Patients with ^18^F-fluorocholine or ^68^Ga prostate-specific membrane antigen ligand positron emission tomography/computed tomography–positive nodal-recurrent prostate cancer and treated with s-IFRT or s-EFRT were retrospectively selected. Time to biochemical failure, time to palliative androgen deprivation therapy (ADT), and distant metastasis–free survival were analyzed.

**Results:**

Between 2009 and 2019, 86 patients were treated with salvage nodal radiation therapy: 38 with s-IFRT and 48 with s-EFRT. After a median follow-up of 41.9 months (5.4-122.1 months), 47 patients presented a further relapse: 31 after s-IFRT and 16 after s-EFRT, with only 1 in-field relapse. The median time to palliative ADT was 24.8 months (95% confidence interval [CI], 13.3-93.5 months) in the s-IFRT group and not yet reached (95% CI, 40.3 months to not yet reached) in the s-EFRT group (*P* = .010). The 3-year biochemical failure–free rate was 70.2% (95% CI, 51.5%-82.9%) with s-IFRT and 73.9% (95% CI, 55.4%-85.7%) with s-EFRT (*P* = .657). The 3-year distant metastasis–free survival was 74.1% (95% CI, 56.0%-85.7%) with s-IFRT and 82.0% (95% CI, 63.0%-91.8%) with s-EFRT (*P* = .338).

**Conclusions:**

s-EFRT and s-IFRT for positron emission tomography–positive nodal-recurrent prostate cancer provide excellent local control. Time to palliative ADT was longer following s-EFRT than following s-IFRT.

## Introduction

Despite the improvement of therapeutic strategies in the management of localized prostate cancer in recent years, 20% to 50% of patients present biochemical failure (BF).^1^, ^2^, ^3^, ^4^, ^5^, ^6^ Conventional imaging (such as computed tomography [CT] scan or bone scintigraphy) performed in cases of BF usually locates recurrences at an already advanced stage, with diffuse nodal or distant progression. The only treatments proposed for these patients are palliative systemic therapies, including androgen deprivation therapy (ADT),^7^ which have substantial side effects that impair quality of life.^8^ New functional imaging has recently been developed in prostate cancer, opening up new therapeutic perspectives. ^18^F-fluorocholine positron emission tomography/CT (FCH PET/CT) and, more recently, ^68^Ga prostate-specific membrane antigen ligand PET/CT (PSMA PET/CT) are increasingly being performed to stage patients with BF after curative treatment for localized prostate cancer. They have better sensitivity and specificity than conventional imaging and make it possible to identify recurrence sites with accuracy at an earlier stage.^9^, ^10^, ^11^, ^12^ The earlier detection of nodal and metastatic relapses, potentially making patients eligible for local salvage treatment, has led to a growing interest to treat these patients with curative intent. Several studies have shown promising results, suggesting that salvage local treatments can delay the initiation of palliative ADT and improve progression-free survival (PFS) with acceptable toxicities.^13^, ^14^, ^15^ However, the modalities for salvage treatment in lymph node (LN) recurrences of prostate cancer are not clearly established^16^^,^^17^ as no published randomized studies to date have assessed different local salvage treatments.

Moreover, as some patients will inevitably progress after individualized salvage treatment, we need better knowledge of the course of the disease in this population in order to properly select patients and to propose the most appropriate therapeutic strategy.

The objective of this study was two-fold. First, we assessed and compared the feasibility and the efficacy of salvage nodal involved-field radiation therapy (s-IFRT) and salvage nodal extended-field radiation therapy (s-EFRT) in patients with FCH or PSMA PET-positive nodal recurrence from prostate cancer. Second, we analyzed the patterns of failure after salvage nodal radiation therapy in this same population.

## Methods and Materials

### Study population

After institutional review board approval, we retrospectively identified patients with nodal recurrence after local curative therapy for prostate cancer, detected with FCH or PSMA PET/CT and treated with salvage radiation therapy with curative intent in our institution.

FCH and PSMA PET/CT were performed as described previously.^18^^,^^19^

### Radiation therapy

An abdominopelvic planning CT scan with 2.5-mm slice thickness was performed starting 5 cm above the diaphragm and ending 2 cm below the ischial tuberosities. Contrast agent was injected in the absence of contraindications. Patients were immobilized in the supine position in a custom blue bag device (VacLok system; CIVCO Medical Solutions, Orange City, IA).

Organs at risk were the rectum in toto, the bladder in toto and the bowel loops (defined as the entire abdominal cavity minus the clinical target volume [CTV]; 2 cm above and below the CTV) and the kidneys.

For s-EFRT, a prophylactic CTV including the whole pelvis was delineated as defined by the Radiation Therapy Oncology Group consensus atlas. In patients with PET-positive LN in the common iliac region or lower para-aortic region, this CTV was extended up to the L2/L3 space.^9^ When lumboaortic (LA) PET-positive LN were involved, the prophylactic CTV was extended up to the renal arteries and a 7-mm margin around the LA vessels anteriorly and laterally (minus bowel loops, bones, and muscles). For the boost to PET-positive LN, gross tumor volume (GTV) was defined as any PET-positive LN delineated after fusion between the planning CT and the CT images from PET/CT. Each CTV was equal to the GTV. A 5-mm margin around the GTV was applied to obtain each planning target volume (PTV). For the bowels, the dose received by 2% of the bowel volume had to be <60 Gy, the mean dose had to be 30 Gy, and the volume of bowels receiving 30 Gy had to be <30%. The prescription to the PTV was expressed in terms of minimum and maximum acceptable dose: 100% of the PTV was covered by the 95% isodose, and no point dose within the PTV could exceed 110%.

For s-IFRT, GTV was defined as any PET-positive LN delineated after fusion between the planning CT and the CT images from PET/CT. Each CTV was equal to the GTV. A 5-mm margin around the GTV was applied to obtain each PTV. Three-dimensional radiation therapy or intensity modulated radiation therapy were used with the same dose constraints as described for s-EFRT. For stereotactic body radiation therapy (SBRT), the absorbed dose to 0.5 cm^3^ of any part of the gastrointestinal (GI) tract had to be ≤30 Gy with a maximum of 36 Gy. For LA LN treated with SBRT, the maximum absorbed doses to the kidneys and spinal cord had to be <12 Gy and <18 Gy, respectively. Treatment was prescribed to the periphery of the PTV (80% of the dose covering 100% of the PTV) and dose distributions were normalized to the isocenter.

The choice of irradiation was determined according to the previous treatments, the characteristics of the patient, and the practices of the physician.

Irradiation was performed on either a TrueBeam, Trilogy, or Novalis linear accelerator equipped with a 120-leaf collimator (Varian Medical Systems, Palo Alto, CA) depending on the technique used. A cone beam CT scan was performed before each fraction for all patients over the entire course of the radiation therapy to set up patients and verify targets; all shifts were corrected with no minimal action level. In each treatment group, all techniques of radiation therapy were included, and the dose prescription was decided at the discretion of the physician.

Treatment characteristics are summarized in Table 1.

**Table 1:** Patients’ and treatment characteristics at the time of PET-positive nodal failure

Characteristic		Total (N = 86)	s-IFRT (n = 38, 44.2%)	s-EFRT (n = 48, 55.8%)	*P* value
Age (y)	Mean (SD)	69.6 (7.5)	70.0 (8.1)	69.3 (7.0)	.676
	Median (range)	70.4 (53.0-85.7)	70.3 (54.6-84.8)	70.4 (53.0-85.7)	
Time from diagnosis of prostate cancer (y)	Mean (SD)	6.4 (4.0)	6.9 (3.9)	6.0 (4.2)	.347
	Median (range)	5.6 (0.3-16.0)	6.5 (1.7-14.7)	5.3 (0.3-16.0)	
PSA value (ng/mL)	Mean (SD)	4.4 (4.4)	5.1 (5.1)	3.8 (3.7)	.061
	Median (range)	3.1 (0.2-29.2)	3.9 (0.4-29.2)	2.5 (0.2-19.0)	
Number of PET-positive LN per patient	Mean (SD)	2.1 (1.8)	1.6 (1.1)	2.5 (2.1)	.012
	Median (range)	1.0 (1.0-12.0)	1.0 (1.0-7.0)	2.0 (1.0-12.0)	
	1 LN	45 (52.3)	24 (63.2)	21 (43.7)	.019
	2 LN	16 (18.6)	9 (23.7)	7 (14.6)	
	3 LN	11 (12.8)	3 (7.9)	8 (16.7)	
	≥4 LN	12 (14)	1 (2.6)	11 (22.9)	
	Missing	2 (2.3)	1 (2.6)	1 (2.1)	
Topography of involved LN per patient	Common iliac	23 (26.7)	10 (26.3)	13 (27)	.672
	Internal iliac	20 (23.2)	7 (18.4)	13 (27)	.202
	External iliac	30 (34.9)	8 (2)	22 (45.8)	.391
	Obturator	10 (11.6)	7 (18.4)	3 (6.25)	.663
	Inguinal	2 (2.3)	1 (2.6)	1 (2.1)	NA
	Lumboaortic	24 (27.9)	9 (23.7)	15 (31.25)	.216
	Mediastinum	1 (1.2)	1 (2.6)	0 (0)	NA
Postoperative salvage RT to prostate bed	Patients, n (%)	12 (14.0)	0 (0.0)	12 (25)	<.001
	Total dose (Gy), median (range)	68.0 (60.0-70.2)	-	68.0 (60.0-70.2)	
	Dose per fraction (Gy), median (range)	2.0 (1.8-2.2)	-	2.0 (1.8-2.2)	
Whole-pelvis irradiation	Patients, n (%)	43 (50.0)	-	43 (89.6)	<.001
	Total dose (Gy), median (range)	46.0 (45.0-54.0)	-	46.0 (45.0-54.0)	
	Dose per fraction (Gy), median (range)	1.8 (1.8-2.2)	-	1.8 (1.8-2.2)	
Lumboaortic irradiation	Patients, n (%)	19 (22.1)	-	19 (39.6)	<.001
	Total dose (Gy), median (range)	46.0 (45.0-59.4)	-	46.0 (45.0-59.4)	
	Dose per fraction (Gy), median (range)	1.8 (1.8-2.0)	-	1.8 (1.8-2.0)	
Salvage RT dose to positive LN	Total dose (Gy), median (range)	-	36.0 (30.0-66.0)	60.0 (18.0-66.6)	<.001
	Dose per fraction (Gy), median (range)	-	7.5 (2.0-15.0)	2.2 (1.8-10.0)	
RT techniques	3D-RT	1 (1.2)	1 (2.6)	0 (0.0)	-
	IMRT/VMAT	36 (41.9)	5 (13.2)	31 (64.6)	
	SIB	15 (17.4)	-	15 (31.3)	
	SBRT	34 (39.5)	32 (84.2)	2* (4.2)	
Concomitant/adjuvant ADT	Patients, n (%)	32 (37.2)	3 (7.9)	29 (60.7)	<.001
Time of ADT (mo)	Mean (SD)	11.7 ± 14.4	3.8 (2.7)	12.6 (14.9)	.149
	Median (range)	5.9 (0.9-58.0)	4.4 (0.9-6.0)	5.9 (2.5-58.0)	

Values are presented as the number (percentage) of patients unless otherwise indicated.

*Abbreviations:* 3D-RT = 3-dimensional radiation therapy; ADT = androgen deprivation therapy; IMRT = intensity modulated radiation therapy; LN = lymph node; NA = not applicable; PET = positron emission tomography; PSA = prostate specific antigen; RT = radiation therapy; s-EFRT = salvage extended-field radiation therapy; s-IFRT = salvage involved-field radiation therapy; SBRT = stereotactic body radiation therapy; SD = standard deviation; SIB = simultaneous integrated boost; VMAT = volumetric modulated arc therapy.

⁎ Both patients were treated with a combination of extended nodal irradiation with IMRT and a nodal SBRT boost.

### Follow-up after salvage nodal radiation therapy

Follow-up was performed as described previously.^18^ For patients with a second clinical relapse after salvage radiation therapy without diffuse metastases, imaging was fused with the planning CT of the salvage radiation therapy. The second relapses were delineated and classified as out of field (if <20% was within the 95% isodose line with normofractionated treatment or within the 80% isodose line with SBRT), marginal (if 20-95% was within the 95% isodose in normofractionated treatment or within the 80% isodose in SBRT) or in field (if ≥95% was within the 95% isodose in normofractionated treatment or within the 80% isodose in SBRT).^20^^,^^21^ Oligometastases were defined as 5 or fewer metastases.

### Statistical analysis

Median follow-up times were calculated using the reverse Kaplan-Meier method.

In this analysis, a BF was defined using the Phoenix definition.^22^

Time to biochemical failure (TTF) was defined as the time between the PET/CT diagnosing the nodal relapse before salvage nodal radiation therapy and the BF. Time to palliative ADT was defined as the time between the PET/CT diagnosing the nodal relapse before salvage nodal radiation therapy and the initiation of palliative life-long ADT. Distant metastasis–free survival (DMFS) was defined as the time between the PET/CT diagnosing the nodal relapse before salvage nodal radiation therapy and distant metastatic progression (including supra-diaphragmatic LN, bone, and visceral metastases) or death.

The outcomes were determined by the Kaplan-Meier method. Hazard ratios (HRs) and 95% confidence intervals (CIs) for univariate and multivariate analyses were estimated using a Cox's proportional hazards regression model with a backward procedure.

The s-IFRT and s-EFRT groups were compared using the Fisher or χ^2^ test for categorical variables and the Student *t* or Mann-Whitney test for quantitative variables (depending on the normality of the distribution).

Statistical analyses were performed using SAS 9.4 software (SAS Institute, Cary, North Carolina).

## Results

### Characteristics of primary disease and at the time of first PET-positive nodal relapses

Between January 2009 and April 2019, 86 patients with BF after primary local therapy for prostate cancer had FCH PET/CT or PSMA PET/CT, on which only nodal relapses were diagnosed (82 using FCH PET/CT and 4 using PSMA PET/CT).

Patients’ characteristics at diagnosis and at the time of first PET-positive nodal relapses are summarized in Tables 1 and 2, respectively.

**Table 2:** Initial patients’ characteristics

Characteristic		Total (N = 86)	s-IFRT (n = 38, 44.2%)	s-EFRT (n = 48, 55.8%)	*P* value
T stage (UICC 2002)	1	15 (17.4)	5 (13.2)	10 (20.8)	.005
	2	40 (46.6)	17 (44.7)	23 (47.9)	
	3	13 (15.1)	11 (28.9)	2 (4.2)	
	Missing	18 (20.9)	5 (13.2)	13 (27.1)	
N stage (UICC 2002)	0	45 (52.3)	17 (44.7)	28 (58.3)	.005
	1	5 (5.8)	0 (0)	5 (10.4)	
	X	36 (41.9)	21 (55.3)	15 (31.3)	
Gleason score	≤6	28 (32.)	17 (44.7)	11 (22.9)	.047
	7	34 (39.6)	14 (36.8)	19 (39.6)	
	≥8	12 (13.9)	2 (5.3)	10 (20.8)	
	Missing	12 (13.9)	5 (13.2)	8 (16.7)	
Baseline PSA value (ng/mL)	Mean (SD)	15.0 (19.6)	18.7 (24.4)	12.1 (14.3)	.309
	Median (range)	9.3 (3.3-129.0)	9.3 (4.0-129.0)	8.8 (3.3-99.7)	
Primary treatments					
Radical prostatectomy		60 (69.8%)	29 (76.3%)	31 (64.6%)	.239
Pelvic lymph node dissection		48 (55.8%)	23 (60.5%)	25 (52%)	.906
Radical prostatectomy followed by postoperative RT		40 (46.5%)	25 (65.8%)	15 (31.3%)	.017
Prostate RT		24 (27.9%)	8 (21%)	16 (33.3%)	.032
Whole pelvic RT		14 (16.3%)	8 (21%)	6 (12.5%)	.590
Brachytherapy*		3 (3.5%)	1 (2.6%)	2 (4.2%)	1
Concomitant/adjuvant ADT		27 (31.4%)	12 (31.6%)	15 (31.3%)	.974

Values are presented as the number (percentage) of patients unless otherwise indicated.

*Abbreviations:* ADT = androgen deprivation therapy; PSA = prostate specific antigen; RT = radiation therapy; SD = standard deviation; s-EFRT = salvage extended-field radiation therapy; S-IFRT = salvage involved-field radiation therapy; UICC = union for international cancer control.

⁎ Boost or exclusive brachytherapy.

At the time of primary disease, 89% of patients were initially treated with exclusive or postoperative radiation therapy in the s-IFRT group and 66% in the s-EFRT group. Twenty-one percent and 12.5% had pelvic irradiation in the s-IFRT group and the s-EFRT group, respectively.

Forty-eight patients were treated with s-EFRT and 38 with s-IFRT. There was a significantly higher number of PET-positive LN in the s-EFRT group than in the s-IFRT group (*P* = .019). In the s-IFRT population, almost 87% of patients had only 1 or 2 positive LN compared with >60% in the s-EFRT group. The topography of involved LN was comparable in the 2 populations, more than a quarter of patients had an extra-pelvic nodal recurrence. Eighty-four percent of patients in the s-IFRT group were treated with SBRT. The median dose per fraction to the PET-positive LN was 7.5Gy. Almost 96% of patients in the s-EFRT group were treated with intensity modulated radiation therapy/volumetric modulated arc therapy techniques including 31% with a simultaneous integrated boost. The median prophylactic total dose to the pelvis was 45 Gy and the median total dose to the PET-positive LN was 60 Gy in the s-EFRT group. Twenty-nine patients in the s-EFRT group and 3 in the s-IFRT group received concomitant/adjuvant ADT.

### Acute and late toxicities of salvage nodal radiation therapy

There was no significant difference between the 2 groups for acute GI and GU toxicities. More than 89% of patients did not experience acute GI and GU toxicities. No grade 3 or more acute GI and GU toxicity was observed.

There was no significant difference between the 2 groups for late GI and GU toxicities. More than 73% of patients did not experience late GI and GU toxicities. One patient had grade 4 late GI and GU in the s-EFRT group.

Acute and late toxicities are reported in detail in Table 3.

**Table 3:** Acute and late toxicities of patients treated with salvage involved-field radiation therapy and salvage extended-field radiation therapy for positron emission tomography–positive nodal recurrences

Toxicity	Total	s-IFRT	s-EFRT	*P* value
Acute GI, n (%)				1
0	80 (93.0)	36 (94.7)	44 (91.7)	
1	3 (3.5)	1 (2.6)	2 (4.2)	
2	3 (3.5)	1 (2.6)	2 (4.2)	
3	0 (0)	-	-	
4	0 (0)	-	-	
Acute GU, n (%)				.314
0	77 (89.5)	34 (89.5)	43 (89.6)	
1	5 (5.8)	1 (2.6)	4 (8.3)	
2	4 (4.7)	3 (7.9)	1 (2.1)	
3	0 (0)	-	-	
4	0 (0)	-	-	
Late GI, n (%)				.275
0	76 (88.4)	32 (84.2)	44 (91.7)	
1	8 (9.3)	5 (13.2)	3 (6.3)	
2	1 (1.2)	1 (2.6)	-	
3	0 (0)	-	-	
4	1 (1.2)	-	1 (2.1)	
Late GU, n (%)				.455
0	63 (73.3)	25 (65.8)	38 (79.2)	
1	8 (9.3)	5 (13.2)	3 (6.3)	
2	12 (14.0)	7 (18.4)	5 (10.4)	
3	2 (2.3)	1 (2.6)	1 (2.1)	
4	1 (1.2)	-	1 (2.1)	

*Abbreviations:* GI = gastrointestinal; GU = genitourinary.

### Time to BF

For the whole population, the median follow-up was 41.9 months (5.4-122.1 months). In the s-IFRT and s-EFRT populations, the median follow-up was 63.2 months (6.2-122.1 months) and 33.8 months (5.4-93.2 months), respectively.

**Fig. 1:**
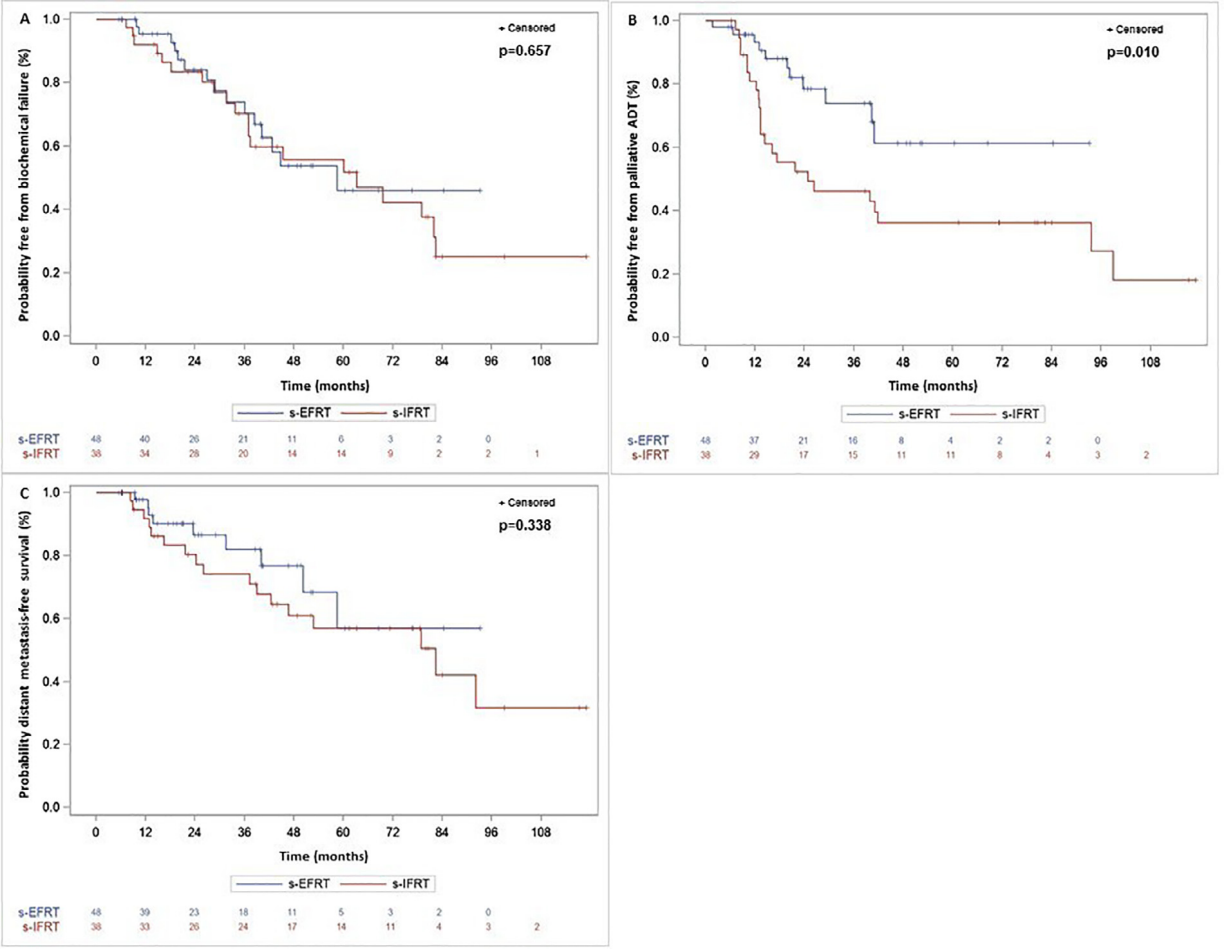
Kaplan Meier analysis of probability in both treatment groups for PET-positive nodal recurrences of biochemical failure (A). Introduction of palliative androgen deprivation therapy (B) and distant metastasis (C). *Abbreviations:* ADT = androgen deprivation therapy; s-EFRT = salvage extended-field radiation therapy; S-IFRT = salvage involved-field radiation therapy.

Overall, 35 patients had a BF after nodal salvage radiation therapy, 20 in the s-IFRT group (52.6%) and 15 in the s-EFRT group (31.3%). For the whole population, the median TTF was 60 months (95% CI, 40.1-82.4 months), and the 3-year BF-free rate was 72.3% (95% CI, 59.8%-81.5%). In the s-IFRT and in the s-EFRT populations, the median TTF was 63.2 months (95% CI, 37-82.4 months) and 58.5 months (95% CI, 38.3 months to not yet reached), respectively, and the 3-year BF-free rate was 70.2% (95% CI, 51.5%-82.9%) and 73.9% (95% CI, 55.4%-85.7%), respectively (*P* = .657). TTF in both groups is presented in Fig. 1A.

Univariate and multivariate analyses are reported in Table 4. Predictive factors for BF were prostate specific antigen (PSA) level at the time of failure >3 ng/mL (HR, 3.23; 95% CI, 1.45-7.18; *P* = .004) and >2 PET-positive LN at the time of failure (HR, 3.65; 95% CI, 1.65-8.05; *P* = .001) in multivariate analysis.

**Table 4:** Univariate and multivariate analyses of biochemical failure–free, palliative androgen deprivation therapy–free, and distant metastasis–free survival

Biochemical failure–free survival						
Variable	Univariate analysis			Multivariate analysis		
	Hazard ratio	95% CI	*P* value	Hazard ratio	95% CI	*P* value
Number of PET-positive LN						
>1 vs ≤1	1.64	0.83-3.25	.152	-	-	-
>2 vs ≤2	2.11	1.00-4.45	.049	3.23 3.25*	1.45-7.18 1.45-7.27	.004
PSA level at time of PET/CT						
>3 vs ≤3 ng/mL	2.90	1.39-6.02	.004	3.65 3.69*	1.65-8.05 1.66-8.20	.001 .001
Radiation therapy modality						
s-EFRT vs s-IFRT	0.86	0.43-1.70	.658	-	-	-
Time between primary diagnosis and PET positive LN						
>5 vs ≤5 y	0.81	0.41-1.60	.552	-	-	-
Concomitant/adjuvant ADT						
Yes vs no	0.90	0.41-1.97	.798	-	-	-

Palliative androgen deprivation therapy–free survival						
Variable	Univariate analysis			Multivariate analysis		
	Hazard ratio	95% CI	*P* value	Hazard ratio	95% CI	*P* value
Number of PET-positive LN						
>1 vs ≤1	1.67	0.84-3.33	.145	-	-	-
>2 vs ≤2	1.48	0.68-3.22	.323	-	-	-
PSA level at time of PET/CT						
>3 vs ≤3 ng/mL	2.77	1.33-5.77	.006	3.46	1.58-7.58	.002
Radiation therapy modality						
s-EFRT vs s-IFRT	0.40	0.19-0.83	.013	0.43	0.20-0.91	.028
Time between primary diagnosis and PET-positive LN						
>5 vs ≤5 y	0.54	0.28-1.06	.073	-	-	-

Distant metastasis–free survival						
Variable	Univariate analysis			Multivariate analysis		
	Hazard ratio	95% CI	*P* value	Hazard ratio	95% CI	*P* value
Number of PET-positive LN						
>1 vs ≤1	2.56	1.16-5.64	.020	-	-	-
>2 vs ≤2	3.31	1.45-7.56	.005	4.33 4.32*	1.80-10.42 1.80-10.30	.001 .001
PSA level at time of PET/CT						
>3 vs ≤3 ng/mL	2.53	1.08-5.95	.033	3.02 2.82*	1.25-7.34 1.16-6.84	.015 .022
Radiation therapy modality						
s-EFRT vs s-IFRT	0.67	0.29-1.53	.341	-	-	-
Time between primary diagnosis and PET-positive LN						
>5 vs ≤5 y	1.12	0.51-2.45	.77	-	-	-
Concomitant/adjuvant ADT						
Yes vs no	0.47	0.16-1.39	.173	-	-	-

*Abbreviations:* ADT = androgen deprivation therapy; CT = computed tomography; LN = lymph node; PET = positron emission tomography; PSA = prostate specific antigen; s-EFRT = salvage extended-field radiation therapy; S-IFRT = salvage involved-field radiation therapy.

⁎ Cox proportional hazards regression after adjusting for ADT.

### Time to palliative ADT

Palliative ADT was introduced after nodal salvage radiation therapy in 35 patients. The median time to palliative ADT for the whole population was 41.9 months (95% CI, 29.1 to not yet reached), the 3-year palliative ADT-free rate was 60.7% (95% CI, 47.9%-71.2%). In the s-IFRT and s-EFRT groups, the median time to palliative ADT was 24.8 months (95% CI, 13.3-93.5 months) and not yet reached (95% CI, 40.3 months to not yet reached), respectively, and the 3-year palliative ADT-free rate was 46.3% (95% CI, 29.3%-61.6%) and 73.8 (95% CI, 54.5%-85.9%), respectively (*P* = .010). Time to palliative ADT following both treatments is presented in Fig. 1B.

The only predictive factor for the initiation of palliative ADT was PSA >3 ng/mL at the time of nodal failure (in multivariate analysis: HR, 3.46; 95% CI, 1.58-7.58; *P* = .002). Patients treated with s-EFRT were less likely to start palliative ADT (in multivariate analysis: HR, 0.43; 95% CI, 0.20-0.91; *P* = .028). Univariate and multivariate analyses are detailed in Table 4.

### DMFS

At last follow-up, 26 patients had distant progression: 17 in the s-IFRT group and 9 in the s-EFRT group. For the whole population, the median DMFS was 82.4 months (95% CI, 52.67 to not yet reached) and the 3-year DMFS was 78.3% (95% CI, 66.3%-86.4%). In the s-IFRT and s-EFRT populations, the median DMFS was 82.4 months (95% CI, 39.0 months to not yet reached) and not yet reached (95% CI, 50.1 months to not yet reached), respectively, and the 3-year DMFS was 74.1% (95% CI, 56.0%-85.7%) and 82.0% (95% CI, 63.0%-91.8%), respectively (*P* = .338). DMFS of both treatment groups is shown in Fig. 1C.

Predictive factors for distant metastasis were >2 PET-positive LN (HR, 4.33; 95% CI, 1.80-10.42; *P* = .001) and PSA >3 ng/mL at the time of nodal failure (HR, 3.02; 95% CI, 1.25-7.34; *P* = .015) in multivariate analysis. Univariate and multivariate analyses are detailed in Table 4.

### Deaths

Six patients died, all of whom were in the s-IFRT group. Two patients died because of the disease; the cause of death for the other 4 patients was unknown.

### Patterns of clinical progression after salvage nodal radiation therapy

Overall, 47 of 86 patients relapsed after salvage radiation therapy: 31 in the s-IFRT group and 16 in the s-EFRT group.

Regarding the second relapse, 33 patients developed clinical relapses. There was 1 local recurrence in the s-IFRT group that was out of field of the nodal salvage radiation therapy. There were 14 pelvic and/or LA nodal recurrence: 12 in the s-IFRT group and 2 in the s-EFRT group. Of the 9 relapses evaluated, all were out of field of the nodal salvage radiation therapy. There was 1 local and LA nodal recurrence in the s-EFRT group. Only the nodal relapse was in the field of nodal salvage radiation therapy. There were 10 distant metastatic recurrences: 3 in the s-IFRT group and 7 in the s-EFRT group (3 patients with supra diaphragmatic LN, 6 bone metastases, and 1 pulmonary metastasis). Seven were oligometastatic and all were out of field of nodal salvage radiation therapy. There were 5 pelvic and/or LA nodal and distant metastatic recurrences: 4 in the s-IFRT group and 1 in the s-EFRT group. Location according to the previous irradiation field was not evaluated because all patients had a diffuse supra diaphragmatic and bone metastatic progression. There were 2 local pelvic and/or LA nodal and distant metastatic recurrence: 1 in the s-IFRT group and 1 in the s-EFRT group. Both patients also presented diffuse supra diaphragmatic and bone metastatic progression.

**Fig. 2:**
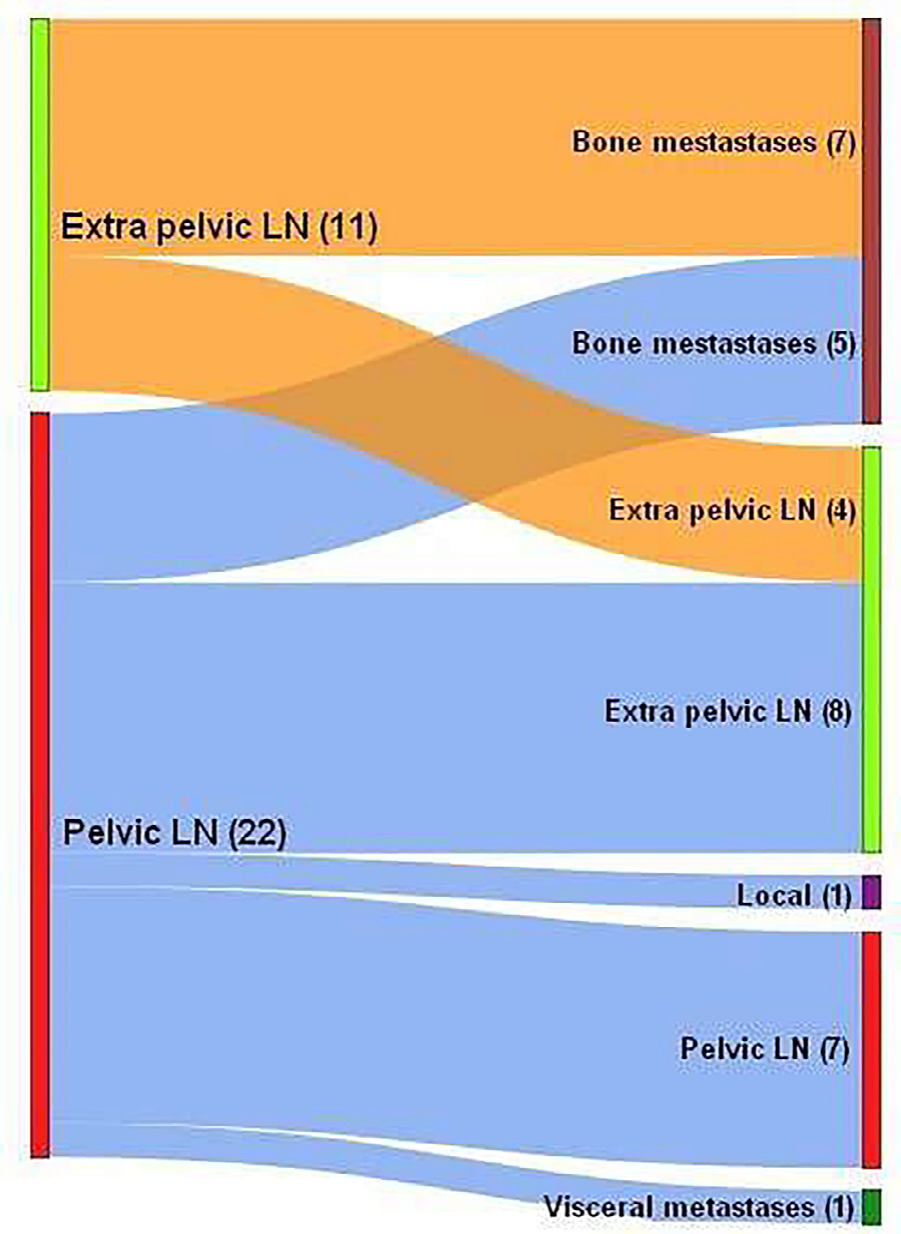
Migration plot showing the relationship between the location of the first nodal relapse treated with salvage extended-field or involved-field radiation therapy (left side) with the location of the second clinical relapse (right side). If a patient presented different relapse locations, he was classified in the most advanced location (defined in ascending order: local, pelvic LN, extra-pelvic LN including lumboaortic LN and supra diaphragmatic LN, bone/visceral metastases). *Abbreviation:* LN = lymph node.

The distribution of clinical second relapses are presented in Fig. 2.

At the second relapse, 9 patients were treated with new salvage radiation therapy (with or without concomitant ADT). One of these patients received a third salvage radiation therapy after new nodal failure.

## Discussion

The emergence of FCH and thereafter PSMA PET/CT has made it possible to detect relapse sites in prostate cancer earlier and led to an evolution in therapeutic strategies in recent years. Different local salvage treatments, also named metastasis-directed therapies (MDT), were assessed to provide local control and to delay palliative ADT.

A randomized phase 2 trial compared the time to the start of palliative ADT following surveillance or MDT (with surgery or SBRT) for PET-positive nodal and/or distant oligorecurrent prostate cancers. The study showed longer ADT-free survival with MDT than with surveillance (median ADT-free survival was 21 months [80% CI, 14-29 months] and 13 months [80% CI, 12-17 months], respectively (HR, 0.60; 80% CI, 0.40-0.90; *P* = .11). Tolerance was good in the MDT group with no grade ≥2 toxicity observed, and quality of life was similar in both groups.^14^

Ost et al also showed a good tolerance of salvage SBRT for FCH PET-positive nodal oligorecurrences with similar PFS,^23^^,^^24^ but more than half of patients had a further relapse after SBRT. Most relapses were in LN. Equivalent results were found with surgical treatments.^25^ In our population, almost 82% of patients treated with s-IFRT relapsed. The most frequent location of relapses following s-IFRT were also in LN. However, none of the nodal relapses were in the irradiation field. These data corroborate others showing that FCH PET/CT misses microscopic disease. Thus, focal salvage treatments, including s-IFRT and nodal dissection, based exclusively on PET-positive LN, seem to be insufficient. The addition of EFRT could potentially delay or even prevent this relapse. Few trials have compared outcomes of s-EFRT (including whole pelvic radiation therapy and a boost of nodal recurrences) with those of s-IFRT in nodal oligorecurrent prostate cancer. In a preliminary analysis, our group showed better TTF with s-EFRT than with s-IFRT (median TTF not yet reached and 39.7 months [95% CI, 10.9 months to not yet reached], respectively; *P* = .009).^18^ With a longer follow-up, our study still showed good outcomes with s-EFRT. Although not significant, patients treated with s-EFRT tended, over time, to have better biochemical TTF and DMFS than did those treated with s-IFRT, but time to palliative ADT was significantly longer with s-EFRT. A larger retrospective multicentric study confirmed fewer nodal recurrences and longer metastasis-free survival after s-EFRT than after SBRT (HR, 0.50; 95% CI, 0.30-0.85; *P* = .009).^13^ The addition of adjuvant pelvic radiation therapy after LN dissection also showed improved PFS.^25^

For BF in prostate cancer, the addition of 6 to 24 months of ADT to postoperative radiation therapy compared with radiation therapy alone for microscopic residual disease led to benefits in PFS and overall survival.^26^^,^^27^ A favorable effect could also be expected in nodal recurrences. The phase 2 OLIGOPELVIS study assessed the feasibility of s-EFRT (54 Gy to the pelvis and 66 Gy to the LN, both 30 fractions) with short-course ADT (6 months) in nodal oligorecurrent prostate cancers.^28^ They showed a relatively good tolerance of the treatment combination.^15^ In our study, concomitant/adjuvant ADT was associated with radiation therapy in 61% of patients in the s-EFRT group versus <8% in the s-IFRT group. We also found similar low toxicity in the 2 groups, thereby confirming the good tolerance of s-IFRT and s-EFRT associated or not with concomitant/adjuvant ADT.

In terms of patterns of failure after salvage nodal radiation therapy, we found an excellent local control and a trend towards a shift in locations. For almost 64% of pelvic LN relapses treated with salvage radiation therapy, the disease progressed to extra-pelvic LN or became metastatic, and almost 64% of extra-pelvic LN relapsed with bone metastases.

PSMA PET/CT performed following BF of prostate cancer showed better detection of relapses with low PSA values. Currently, very few data concerning MDT guided specifically with PSMA PET/CT are available. No interpretation could be given in our study; there were only 4 nodal relapses diagnosed with PSMA PET/CT, and 1 of these presented diffuse progression. In a retrospective study that assessed patients exclusively treated with PSMA PET/CT-guided radiation therapy for recurrent oligometastatic prostate cancer, 59% of oligorecurrences were LN and were treated with an extended irradiation field. The authors showed that salvage radiation therapy for PSMA PET/CT-positive oligometastases resulted in effective local control with prolonged biochemical PFS (median of 22 months [95% CI, 20.2-24.0 months]). They also reported a shift in new progressions towards distant LN and skeletal metastases.^29^

So far, most published studies on MDT, including those discussed here, have been small, retrospective studies with heterogeneous populations, and have included distant and nodal recurrences. As a result, they cannot be used to establish the optimal management for nodal only oligorecurrent prostate cancers.^13^ Further specific prospective studies are needed. Currently there are 2 ongoing randomized trials. The European PEACE V study is a randomized phase 2 trial assessing the impact of adding whole pelvic radiation therapy to MDT (salvage lymph node dissection or SBRT) associated with 6 months of ADT in oligorecurrent nodal prostate cancers. The primary endpoint is metastasis-free survival.^30^ OLIGOPELVIS 2 is a French randomized phase 3 trial based on the hypothesis that salvage pelvic radiation therapy may prolong the interval between the first and second intermittent ADT in nodal oligorecurrent prostate cancer. The authors are comparing intermittent ADT (6 months) alone with intermittent ADT associated with s-EFRT.^28^ In these 2 trials, nodal recurrences will be detected with FCH or PSMA PET/CT.

## Conclusion

Our study showed the feasibility of s-IFRT and s-EFRT for PET-positive nodal-recurrent prostate cancer with excellent local control. Time to the initiation of palliative ADT was longer following S-EFRT than following s-IFRT.
